# Original research Socio-demographic patterning of self-reported physical activity and sitting time in Latin American countries: findings from ELANS

**DOI:** 10.1186/s12889-019-8048-7

**Published:** 2019-12-23

**Authors:** Gerson Luis de Moraes Ferrari, Irina Kovalskys, Mauro Fisberg, Georgina Gómez, Attilio Rigotti, Lilia Yadira Cortés Sanabria, Martha Cecilia Yépez García, Rossina Gabriella Pareja Torres, Marianella Herrera-Cuenca, Ioná Zalcman Zimberg, Viviana Guajardo, Michael Pratt, Abby C. King, Dirceu Solé, on behalf of the ELANS Study Group, Mauro Fisberg, Irina Kovalskys, Georgina Gómez, Attilio Rigotti, Lilia Yadira Cortés Sanabria, Martha Cecilia Yépez García, Rossina Gabriella Pareja Torres, Marianella Herrera-Cuenca, Berthold Koletzko, Luis A. Moreno, Michael Pratt, Regina Mara Fisberg, Viviana Guajardo, Ioná Zalcman Zimberg, María Paz Amigo, Ximena Janezic, Natasha Aparecida Grande de França, Guadalupe Echeverría, Leslie Landaeta, Óscar Castillo, Luz Nayibe Vargas, Luisa Fernanda Tobar, Yuri Milena Castillo, Rafael Monge Rojas, Mónica Villar Cáceres, María Belén Ocampo, Rossina Pareja Torres, María Reyna Liria, Krysty Meza, Maritza Landaeta, Betty Méndez, Maura Vasquez, Omaira Rivas, Carmen Meza, Servando Ruiz, Guillermo Ramirez, Pablo Hernández, Priscila Bezerra Gonçalves, Claudia Alberico, Gerson Luis de Moraes Ferrari

**Affiliations:** 1 Centro de Investigación en Fisiologia del Ejercicio – CIFE, Universidad Mayor, José Toribio Medina, 29. Estacion Central, Santiago, Chile; 2 Departamento de Pediatria da Universidade Federal de São Paulo, São Paulo, Brazil; 3 Commitee of Nutrition and Wellbeing, International Life Science Institute (ILSI-Argentina), Buenos Aires, Argentina; 4 Instituto Pensi, Fundação José Luiz Egydio Setubal, Hospital Infantil Sabará, São Paulo, Brazil; 5 Departamento de Bioquímica, Escuela de Medicina, Universidad de Costa Rica, San José, Costa Rica; 6 Centro de Nutrición Molecular y Enfermedades Crónicas, Departamento de Nutrición, Diabetes y Metabolismo, Escuela de Medicina, Pontificia Universidad Católica, Santiago, Chile; 7 Departamento de Nutrición y Bioquímica, Pontificia Universidad Javeriana, Bogotá, Colombia; 8 Colégio de Ciencias de la Salud, Universidad San Francisco de Quito, Quito, Ecuador; 9 Instituto de Investigación Nutricional, La Molina, Lima, Peru; 10 Centro de Estudios del Desarrollo, Universidad Central de Venezuela (CENDES-UCV)/Fundación Bengoa, Caracas, Venezuela; 11 Departamento de Psicobiologia, Universidade Federal de São Paulo, São Paulo, Brazil; 12 Institute for Public Health, University of California San Diego, La Jolla, CA USA; 13 Health Research & Policy Department and the Stanford Prevention Research Center, Department of Medicine, Stanford University School of Medicine, Stanford, CA USA

**Keywords:** Epidemiology, Physical activity, Sitting time, Self-report, Public health

## Abstract

**Background:**

Low levels of physical activity (PA) and prolonged sitting time (ST) increase the risk of non-communicable diseases and mortality, and can be influenced by socio-demographic characteristics. The aim of this study was to use self-report data to characterise socio-demographic patterns of PA and ST in eight Latin American countries.

**Methods:**

Data were obtained from the Latin American Study of Nutrition and Health (ELANS), a household population-based, multi-national, cross-sectional survey (*n* = 9218, aged 15–65 years), collected from September 2014 to February 2015. Transport and leisure PA and ST were assessed using the International Physical Activity Questionnaire–long version. Overall and country-specific mean and median levels of time spent in transport and leisure PA and ST were compared by sex, age, socioeconomic and education level.

**Results:**

Mean levels of transport and leisure PA were 220.3 min/week (ranging from 177.6 min/week in Venezuela to 275.3 min/week in Costa Rica) and 316.4 min/week (ranging from 272.1 min/week in Peru to 401.4 min/week in Ecuador). Transport and leisure PA were higher (*p* < 0.005) in men than women with mean differences of 58.0 and 34.0 min/week. The mean and median for transport PA were similar across age groups (15–29 years: mean 215.5 and median 120 min/week; 30–59 years: mean 225.0 and median 120 min/week; ≥60 years: mean 212.0 and median 120 min/week). The median time spent in transport and leisure PA between three strata of socioeconomic and education levels were similar. The prevalence of not meeting PA recommendations were 69.9% (95% CI: 68.9–70.8) for transport and 72.8% (95% CI: 72.0–73.7) for leisure. Men, younger people (15–29 years), individuals with higher socioeconomic and education levels spent significantly (*p* < 0.001) more time sitting than women, older people (30–59 years and ≥ 60 years) and those in the middle and low socioeconomic and education groups, respectively.

**Conclusions:**

Transport and leisure PA and ST range widely by country, sex, and age group in Latin America. Programs for promoting leisure and transport PA and reducing ST in Latin America should consider these differences by age and gender and between countries.

**Trial registration:**

ClinicalTrials.Gov NCT02226627. Retrospectively registered on August 27, 2014.

## Introduction

The incidence of cardiovascular diseases (CVD) is increasing throughout the developing world; causing more than 16 million deaths each year, 80% of which occur in low and middle-income countries [1]. Regular physical activity reduces the risk of cardiovascular mortality [2]. Physical inactivity also [3] accounts for 1–3% of health care costs, excluding costs associated with mental health and musculoskeletal conditions [4] and contributes 6% of the mortality burden of coronary heart disease and 10% of breast and colon cancer [5].

Sedentary behavior, as distinct from physical activity, encompasses a broad range of behaviors that involve a sitting or reclining posture and do not increase energy expenditure above 1.5 metabolic equivalents during waking time [6]. Sedentary behaviours are associated with CVD, cancer and all-cause mortality, independent of physical activity [7]. Current physical activity guidelines do not prescribe a quantitative guideline for sitting time [4, 8].

On the basis of this evidence the World Health Organization (WHO) has developed global recommendations for physical activity and an action plan for increasing physical activity and decreasing time spent in sedentary behavior [4]. Policy development and evaluation in this area depend on consistent and valid assessment of prevalence and trends in physical activity, adherence to physical activity recommendations, and time spent sitting. Continued improvements in monitoring physical activity and sitting time are needed to guide policy making and programs for increasing physical activity and reducing sitting time [4].

In recent decades, Latin America has experienced accelerated demographic and epidemiological transitions, and many countries are facing the double burden of communicable and non-communicable diseases [9]. In Latin America physical inactivity levels are high, national health care expenditures due to inactivity are more than USD 3 billion [10], and inactivity has been identified as a critical public health challenge [9, 11], Evidence from a comprehensive review suggests that populations with higher levels of transport physical activity have higher overall levels of physical activity than those populations who rely more on private transportation [12, 13], and that individuals who engage in transport physical activity have lower risk of CVD and all-cause mortality [13, 14]. However, data on physical activity by mode (e.g., active transportation, leisure activity, sitting time) and country in Latin America remain scarce [15]. As such, international comparisons are difficult [16]. Latin America is the most urbanized region in the world, with nearly 80 % of people living in cities [17]. At the same time, the urban environment in Latin America differs considerably from those in the high-income countries [18] and has the largest percentage of the population living in slums [17], and high rates crime and violence [19]. Physical activity and sitting patterns vary by sociodemographic characteristics such as country, sex, age, level of income, and education [15] and these factors must be taken into account as public health programs are developed. The aim of this study was to use self-report data to characterise socio-demographic patterns of physical activity and sitting time in eight Latin American countries in order to better inform public health policy and programs in the region.

## Methods

### Latin American Study of Nutrition and Health and sample

The Latin American Study of Nutrition and Health (*Estudio Latinoamericano de Nutrición y Salud*; ELANS) is a cross-sectional, multinational representative sample conducted in 8 of the 33 Latin America countries (Argentina, Brazil, Chile, Colombia, Costa Rica, Ecuador, Peru and Venezuela). Only urban populations were included to enhance comparability across countries and for reasons of feasibility [20]. The present study used large-scale urban population samples, and these prevalence estimates may reasonably be generalized to the country level given the high degree of urbanization. Data were collected from September 2014 to February 2015. The overarching ELANS protocol was approved by the Western Institutional Review Board (#20140605) and is registered at ClinicalTrials.gov (#NCT02226627). Additional site-specific protocols were also approved by the ethical review boards of the participating institutions. All of the participants provided informed consent/assent for participation in their country-level study. The eight participating countries followed a common protocol, including training for all research professionals. A balance number of participants were stratified by sex, age group and socioeconomic level. In total, 9218 (4409 [47.8%] men) participants aged 15–65 years were included in the study. Sample size and exclusion criteria can be found elsewhere [21].

### Physical activity and sitting time assessment

Physical activity and sitting time were assessed using a Spanish language long-form “last 7 days” self-administered version of the International Physical Activity Questionnaire (IPAQ) [22]. The IPAQ contains questions about the amount of walking, moderate physical activity, and vigorous physical activity occurring as part of active transport and in leisure-time [22]. The transport and leisure-time physical activity sections were included, due to the greater relevance of these domains for guiding public health policies and programs [23], and the poor validity of the IPAQ occupational and home-based physical activity questions in Latin American urban settings.

Data were analyzed in accordance with the IPAQ scoring protocol (www.ipaq.ki.se). IPAQ assesses walking separately. Thus, IPAQ physical activity data are reported as min/day of walking, moderate, and vigorous physical activity. Total time (min/week) and time spent in each of the physical activity modes (i.e., transport and leisure-time) were estimated and used as analysis variables. We analyzed transport physical activity (walking + bicycle) and leisure physical activity (walking + moderate + vigorous) separately. In addition, the IPAQ contained two items capturing sitting time. Sitting time was assessed from the questions in the IPAQ long-form [22, 24]. Participants were asked to report time spent sitting over the past 7 days, with separate amounts reported for weekdays and weekends. We calculated average sitting time per day (min/day) as follows: (weekday time*5 + weekend time*2)/7 [25].

### Sociodemographic characteristics

Participants reported age by year (15–65 years), and three age groups (15–29, 30–59, and ≥ 60 years) were defined to ensure adequate sample sizes. Sex, socioeconomic and educational level were categorized using standard questionnaires. Socioeconomic level was evaluated by questionnaire using country-specific definitions based on national norms, laws, and the questionnaires used on national surveys in each country [26–31]. Given the variability in categorizing socioeconomic strata, a standard three level system (low, medium, high) was developed [21]. A similar process was used to standardize level of education in three strata (basic or lower [low], high school [medium], and university degree [high]) in the eight countries.

### Statistical analysis

Descriptive analyzes are presented as arithmetic mean, median, frequency, percentage, and 95% confidence intervals (95% CI) for physical activity (transport and leisure time) and sitting time for each country and for the entire sample (sum of the eight countries). Since minutes of physical activity (transport and leisure time) and sitting time were not normally distributed, values for the 25th, 50th and 75th percentile were also obtained. A Kolmogorov-Smirnov test was applied to evaluate the data distribution. Differences between groups were analyzed using Wilcoxon or Kruskal-Wallis tests.

The main outcomes were the mean and median time, in min/week, spent in the two modes of physical activity (transport: walking + bicycle; leisure: walking + moderate + vigorous) and sitting time (min/day). Results were stratified by sex, age group, socioeconomic level, and educational level. We also reported the proportion of each group meeting the WHO physical activity guidelines (e.g. > 60 min/day for adolescents and > 150 min/week of moderate-to-vigorous physical activity for adults) in transport and leisure. Data analyses were performed with IBM SPSS, version 22 for Windows [32]. The samples were weighted to adjust for sociodemographic characteristics, sex, and income [21].

## Results

The proportion of women (52.2%; 95% CI: 51.2–53.2) was higher than men (47.8%; 95% CI: 46.8–48.8). In terms of age, 39.4% (95% CI: 38.5–40.5) of participants were aged < 30 years, 53.9% (95% CI: 52.9–55.0) aged 30–59 years, and 6.7% (95% CI: 6.1–7.2) aged ≥60 years. About half were classified as having a low socioeconomic level (52.0%; 95% CI: 51.0–53.0) and/or low educational level (61.2%; 95% CI: 60.3–62.3) (Table 1).

**Table 1:** Sample distribution (%) according to sex, age group, socioeconomic level, and educational level from ELANS study

Country	N	Sex		Age group (years)			Socioeconomic level ^a^			Educational level		
		Men	Women	15–29	30–59	≥60	Low	Medium	High	Low	Medium	High
Argentina	1266	45.3	54.7	35.9	56.4	7.7	48.7	46.2	5.1	75.4	20.3	4.3
Brazil	2000	47.1	52.9	35.6	57.2	7.2	45.8	45.8	8.4	48.4	43.2	8.4
Chile	879	48.4	51.6	38.3	54.9	6.8	46.8	44.1	9.1	65.1	23.7	11.2
Colombia	1230	49.0	51.0	39.3	52.7	8.0	63.3	31.2	5.4	65.0	23.9	11.1
Costa Rica	798	49.4	50.6	41.4	53.6	5.0	32.8	53.6	13.6	81.6	12.6	5.8
Ecuador	800	49.6	50.4	43.0	51.1	5.9	49.9	37.1	13.0	83.0	10.5	6.5
Peru	1113	47.0	53.0	44.2	50.5	5.3	47.9	31.9	20.2	23.1	67.1	9.8
Venezuela	1132	48.8	51.2	42.4	51.7	5.9	77.7	16.8	5.5	68.6	12.6	18.8
Total	9218	47.8	52.2	39.4	53.9	6.7	52.0	38.4	9.6	61.2	29.3	9.5

^a^Estimate distribution of sample (n) according to socioeconomic level

Overall, the response rate for IPAQ was 99.4%. For transport physical activity, Venezuela had the lowest values (mean: 177.6 min/week; 95% CI: 160.7–194.6; median: 100.0 min/week), and the highest average was in Costa Rica (mean: 275.3 min/week; 95% CI: 249.6–301.8; median: 147.0 min/week). The difference between these two countries was 97.7 min/week. For leisure physical activity, the highest values were in Ecuador (mean: 401.4 min/week; 95% CI: 370.6–435.3; median: 240.0 min/week) and the lowest was in Peru (mean: 272.1 min/week; 95% CI: 248.1–297.3; median: 150.0 min/week), with a mean difference of 129.3 min/week between these two countries. For sitting time, the mean difference between Argentina (highest sitting time) and Ecuador (lowest sitting time) was 196.3 min/day (Table 2).

**Table 2:** Characteristics of participants by transport, leisure physical activity and sitting time by sex from ELANS study

Country	Total		Men		Women		*P value*
	Mean (95% CI)	Median (Q1-Q3)	Mean (95% CI)	Median (Q1-Q3)	Mean (95% CI)	Median (Q1-Q3)	
Transport physical activity (min/week)							
Argentina	251.9 (231.7–271.7)	140.0 (60.0–300.0)	288.2 (247.6–328.7)	130.0 (60.0–350.0)	227.4 (204.4–205.0)	140.0 (60.0–280.0)	0.702
Brazil	206.2 (192.1–220.8)	105.0 (60.0–210.0)	245.9 (220.5–271.9)	120.0 (60.0–245.0)	170.1 (155.6–185.6)	105.0 (60.0–210.0)	0.008
Chile	216.7 (194.3–238.9)	105.0 (60.0–105.0)	262.4 (222.8–301.8)	137.5 (70.0–280.0)	173.8 (152.3–194.3)	100.0 (50.0–210.0)	<0.001
Colombia	231.0 (212.4–251.3)	120.0 (25.0–240.0)	277.4 (243.5–307.9)	140.0 (71.5–295.5)	188.5 (166.5–210.2)	105.0 (50.0–210.0)	<0.001
Costa Rica	275.3 (249.6–301.8)	147.0 (63.0–350.0)	308.4 (268.6–351.9)	180.0 (70.0–400.0)	244.8 (217.2–277.4)	140.0 (60.0–283.7)	0.087
Ecuador	221.3 (201.1–242.9)	140.0 (75.0–210.0)	250.5 (221.6–284.7)	140.0 (75.0–300.0)	191.5 (167.6–214.1)	120.0 (70.0–210.0)	0.404
Peru	194.4 (179.8–209.4)	120.0 (60.0–210.0)	204.9 (182.5–228.2)	120.0 (60.0–210.0)	185.3 (167.7–203.8)	120.0 (60.0–210.0)	0.618
Venezuela	177.6 (160.7–194.6)	100.0 (50.0–180.0)	176.3 (148.9–205.3)	90.0 (50.0–175.0)	178.7 (156.5–201.2)	100.0 (50.0–200.0)	0.599
Overall	220.3 (213.5–227.0)	120.0 (60.0–240.0)	251.2 (238.9–262.2)	125.0 (60.0–280.0)	193.2 (185.2–200.5)	105.0 (60.0–210.0)	<0.001
Leisure physical activity (min/week)							
Argentina	298.1 (271.7–327.4)	180.0 (90.0–390.0)	295.7 (259.7–335.4)	180.0 (90.0–390.0)	300.7 (262.3–341.0)	205.0 (90.0–378.7)	0.505
Brazil	312.6 (285.7–339.8)	180.0 (70.0–420.0)	345.9 (308.1–384.9)	180.0 (90.0–486.0)	273.8 (240.9–310.9)	140.0 (60.0–360.0)	0.018
Chile	341.4 (306.7–378.5)	210.0 (90.0–420.0)	360.5 (310.5–416.1)	210.0 (85.0–445.0)	322.3 (280.3–369.1)	210.0 (90.0–408.7)	0.817
Colombia	290.6 (265.2–316.6)	150.0 (60.0–360.0)	312.4 (276.8–348.4)	180.0 (90.0–403.7)	263.5 (226.1–307.7)	130.0 (60.0–300.0)	0.025
Costa Rica	293.2 (259.0–329.6)	180.0 (70.0–360.0)	330.4 (282.5–386.3)	180.0 (90.0–420.0)	249.6 (207.2–290.7)	150.0 (60.0–300.0)	0.025
Ecuador	401.4 (370.6–435.3)	240.0 (131.2–480.0)	358.9 (322.7–399.1)	240.0 (120.0–480.0)	447.6 (394.5–502.5)	280.0 (140.0–577.5)	0.064
Peru	272.1 (248.1–297.3)	150.0 (60.0–355.0)	293.9 (259.9–327.9)	180.0 (80.0–370.0)	246.4 (210.6–284.8)	120.0 (60.0–300.0)	0.013
Venezuela	333.5 (296.9–376.3)	227.5 (105.0–438.5)	393.6 (333.4–448.9)	255.0 (120.0–540.0)	244.7 (202.1–289.3)	180.0 (81.7–300.0)	0.014
Overall	316.4 (306.1–327.1)	180.0 (90.0–420.0)	332.3 (317.5–347.5)	198.0 (90.0–440.0)	298.3 (281.7–314.6)	180.0 (60.0–375.0)	0.011
Sitting time (min/day)							
Argentina	540.7 (526.0–557.9)	480.0 (327.5–720.0)	548.4 (524.3–572.7)	480.0 (330.0–720.0)	534.4 (514.4–553.1)	480.0 (300.0–720.0)	0.971
Brazil	433.9 (420.3–448.1)	360.0 (210.0–600.0)	462.3 (441.2–484.4)	420.0 (240.0–636.0)	408.8 (391.3–427.6)	360.0 (180.0–598.0)	0.003
Chile	485.8 (466.2–499.1)	420.0 (300.0–660.0)	497.5 (470.4–527.4)	450.0 (300.0–660.0)	474.6 (449.1–500.8)	420.0 (300.0–600.0)	0.387
Colombia	481.9 (466.2–499.1)	420.0 (240.0–660.0)	507.3 (482.7–532.9)	480.0 (270.0–720.0)	457.5 (435.3–482.7)	420.0 (239.0–658.0)	0.079
Costa Rica	452.3 (431.3–474.3)	389.0 (240.0–600.0)	486.9 (454.5–520.8)	420.0 (240.0–660.0)	418.1 (394.0–444.7)	360.0 (210.0–570.0)	0.369
Ecuador	344.3 (328.1–362.1)	300.0 (180.0–480.0)	486.8 (454.6–520.8)	300.0 (180.0–500.0)	321.5 (299.6–344.7)	270.0 (180.0–430.0)	0.076
Peru	525.9 (508.3–544.0)	480.0 (328.0–690.0)	548.4 (522.5–572.8)	510.0 (360.0–720.0)	506.1 (482.7–529.4)	480.0 (300.0–660.0)	0.098
Venezuela	383.3 (367.1–398.5)	360.0 (180.0–540.0)	389.4 (367.2–413.3)	360.0 (180.0–540.0)	377.4 (355.3–396.9)	360.0 (180.0–500.0)	0.079
Overall	460.2 (453.8–466.5)	420.0 (240.0–600.0)	479.1 (470.4–488.0)	420.0 (240.0–660.0)	442.7 (434.6–450.8)	418.0 (240.0–600.0)	<0.001

95% CI: 95% confidence interval

The levels of transport physical activity were higher for men (mean: 251.2 min/week; 95% CI: 238.9–262.2; median: 125.0 min/week) than for women (mean: 193.2 min/week; 95% CI: 185.2–210.0; median: 105.0 min/week) overall, with a mean difference of 58 min/week. The largest sex difference was in Colombia (88.9 mean min/week), followed by Chile (88.6 mean min/week) and the smallest sex difference was in Venezuela (2.4 mean min/week). For leisure physical activity, the largest sex difference was in Venezuela (148.9 mean min/week) and the smallest was in Argentina (5 mean min/week). Men (mean: 479.1 min/day; 95% CI: 470.4–488.0; median: 420.0 min/day) spent more time sitting than women (442.7 min/day; 95% CI: 434.6–450.8; median: 418.0 min/day). The mean difference between sex was 36.4 mean min/day. In overall, men had a significantly higher values than women for transport (*p* < 0.001), leisure (*p* = 0.011) physical activity and sitting time (*p* < 0.001).

Overall, the mean and median transport physical activity were similar across age groups (15–29 years: mean 215.5 min/week, 95% CI: 205.0–226.7 and median 120 min/week; 30–59 years: mean 225.0 min/week, 95% CI: 215.7–234.3 and median 120 min/week; ≥60 years: 212.0 min/week, 95% CI: 187.1–238.0 and median 120 min/week). Significant difference (*p* = 0.027) between age group for transport physical activity was found only in the Ecuador (Table 3).

**Table 3:** Characteristics of participants by transport, leisure physical activity and sitting time by age group from ELANS study

Country	15–29 years		30–59 years		≥60 years		*P value*
	Mean (95% CI)	Median (Q1-Q3)	Mean (95% CI)	Median (Q1-Q3)	Mean (95% CI)	Median (Q1-Q3)	
Transport physical activity (min/week)							
Argentina	237.2 (205.4–270.2)	120.0 (60.0–290.0)	254.9 (227.2–286.3)	140.0 (60.0–300.0)	304.9 (217.9–407.9)	132.5 (60.0–330.0)	0.652
Brazil	213.6 (189.2–236.9)	105.0 (60.0–210.0)	207.4 (190.2–227.7)	120.0 (60.0–210.0)	156.6 (117.5–209.3)	90.0 (60.0–210.0)	0.247
Chile	216.1 (186.9–249.7)	120.0 (70.0–240.0)	210.8 (179.5–242.2)	105.0 (60.0–210.0)	269.0 (149.3–407.4)	105.0 (41.2–210.0)	0.609
Colombia	218.0 (190.7–247.2)	105.0 (60.0–234.7)	246.3 (216.6–275.8)	120.0 (60.0–270.0)	196.0 (147.8–249.8)	105.0 (60.0–228.0)	0.796
Costa Rica	288.5 (250.1–327.9)	160.0 (75.0–360.0)	271.9 (238.1–307.2)	140.0 (60.0–345.0)	204.0 (123.4–301.3)	82.5 (45.0–240.0)	0.180
Ecuador	195.4 (168.3–224.3)	120.0 (70.0–210.0)	241.6 (210.2–272.4)	140.0 (75.0–256.2)	238.1 (178.7–312.6)	165.0 (101.2–295.0)	0.027
Peru	184.9 (162.4–209.0)	105.0 (60.0–210.0)	199.1 (179.8–220.8)	120.0 (60.0–210.0)	226.7 (166.1–308.6)	150.0 (70.0–227.5)	0.088
Venezuela	182.2 (155.4–212.3)	100.0 (60.0–180.0)	180.1 (154.8–208.1)	90.0 (50.0–205.0)	124.2 (94.2–158.4)	95.0 (47.2–150.0)	0.533
Overall	215.5 (205.0–226.7)	120.0 (60.0–210.0)	225.0 (215.7–234.3)	120.0 (60.0–240.0)	212.0 (187.1–238.0)	120.0 (60.0–210.0)	0.384
Leisure physical activity (min/week)							
Argentina	329.2 (288.2–376.2)	240.0 (90.0–450.0)	274.7 (238.3–314.6)	180.0 (80.0–360.0)	262.6 (182.4–371.3)	180.0 (90.0–326.2)	0.041
Brazil	358.8 (314.5–405.2)	232.5 (90.0–480.0)	270.1 (240.2–300.3)	146.0 (60.0–360.0)	363.5 (230.4–518.0)	180.0 (80.0–420.0)	0.011
Chile	382.2 (331.5–438.5)	240.0 (120.0–480.0)	297.0 (252.4–342.3)	180.0 (60.0–384.0)	402.5 (247.2–578.4)	195.0 (60.0–645.0)	0.057
Colombia	313.3 (270.2–360.5)	170.0 (60.0–420.0)	273.6 (240.6–310.6)	150.0 (60.0–322.5)	267.9 (174.6–379.8)	169.5 (60.0–310.0)	0.711
Costa Rica	307.1 (255.1–364.5)	160.0 (60.0–360.0)	280.3 (237.7–322.2)	180.0 (90.0–350.0)	285.2 (137.4–455.2)	160.0 (70.0–270.0)	0.969
Ecuador	380.6 (339.7–428.1)	255.0 (123.7–480.0)	412.2 (362.9–462.8)	240.0 (121.2–507.5)	470.8 (334.1–604.1)	370.0 (150.0–622.5)	0.578
Peru	296.2 (260.9–332.9)	165.0 (70.0–360.0)	252.6 (220.9–288.3)	140.0 (60.0–302.5)	191.6 (127.3–274.8)	135.0 (65.0–240.0)	0.138
Venezuela	395.8 (339.2–458.2)	300.0 (120.0–520.0)	266.1 (213.9–322.4)	180.0 (72.5–357.5)	178.1 (94.5–296.8)	147.0 (60.0–180.0)	0.001
Overall	342.1 (326.0–359.9)	210.0 (90.0–450.0)	292.2 (278.0–305.9)	180.0 (70.0–360.0)	315.4 (269.8–358.1)	180.0 (80.0–390.0)	<0.001
Sitting time (min/day)							
Argentina	544.1 (516.9–569.3)	480.0 (360.0–720.0)	534.9 (514.6–555.1)	480.0 (300.0–720.0)	569.4 (507.8–633.5)	570.0 (307.0–720.0)	0.745
Brazil	493.0 (467.6–519.7)	420.0 (240.0–660.0)	403.8 (386.7–420.7)	360.0 (180.0–540.0)	385.4 (342.5–429.8)	360.0 (185.0–500.0)	<0.001
Chile	539.2 (509.5–569.9)	480.0 (360.0–720.0)	452.2 (428.0–479.1)	420.0 (270.0–570.0)	453.3 (390.7–526.6)	420.0 (245.0–600.0)	<0.001
Colombia	536.9 (508.3–566.7)	480.0 (300.0–720.0)	442.8 (419.8–468.7)	390.0 (238.0–600.0)	473.3 (415.6–540.2)	392.5 (240.0–615.0)	<0.001
Costa Rica	503.7 (468.6–538.9)	465.0 (240.0–690.0)	420.1 (393.2–448.7)	360.0 (210.0–558.7)	365.9 (292.5–440.7)	300.0 (195.0–480.0)	<0.001
Ecuador	362.2 (336.9–391.3)	300.0 (180.0–510.0)	333.7 (310.8–360.6)	300.0 (165.0–450.0)	303.0 (240.5–363.0)	240.0 (170.0–450.0)	0.293
Peru	556.8 (530.3–585.2)	540.0 (360.0–720.0)	503.9 (480.9–528.8)	480.0 (300.0–660.0)	477.1 (411.8–541.6)	465.0 (318.7–600.0)	0.008
Venezuela	408.9 (383.9–433.6)	360.0 (240.0–540.0)	366.2 (344.9–388.1)	360.0 (180.0–490.0)	349.2 (295.7–412.7)	300.0 (135.0–480.0)	0.099
Overall	496.8 (486.5–507.3)	475.0 (270.0–660.0)	436.8 (428.7–444.6)	390.0 (240.0–600.0)	432.6 (410.6–455.3)	360.0 (240.0–600.0)	<0.001

95% CI: 95% confidence interval

The time spent in leisure physical activity among the 15–29 years old group was significantly higher in Brazil (*p* = 0.011), Venezuela (*p* = 0.001) and in overall (*p* < 0.001) than for those 30–59 and ≥ 60 years old, and the higher difference between 15 and 29 and ≥ 60 years was in Venezuela (217.7 mean min/week). For sitting time, the time spent was significantly higher in 15–29 years old than for those 30–59 and ≥ 60 years in overall and in five countries (Brazil, Chile, Colombia, Costa Rica, and Peru (Table 3).

In each country and overall, the median times spent in transport physical activity were similar between the three socioeconomic strata (*p* > 0.05). In leisure physical activity, we found significant differences in Brazil (low: mean 293.0 min/week, 95% CI: 252.4–336.3 and median 140.0 min/week; medium: mean 332.8 min/week, 95% CI: 295.4–371.9 and median 230 min/week; high: mean 307.6 min/week, 95% CI: 239.1–381.9 and median 180 min/week) and in Peru (low: mean 344.7 min/week, 95% CI: 299.5–391.2 and median 139.0 min/week; medium: mean 268.7 min/week, 95% CI: 226.6–312.3 and median 180.0 min/week; high: mean 281.6 min/week, 95% CI: 234.5–333.1 and median 120 min/week) between strata socioeconomic level. The results do not show a clear association between socioeconomic level and transport or leisure physical activity. There are no significant differences between socioeconomic levels for transport and leisure physical activity. Overall, individuals with higher socioeconomic (*p* < 0.001) and education levels (*p* < 0.001) spent more time sitting than those in the middle and low socioeconomic and education groups (Tables 4 and 5).

**Table 4:** Characteristics of participants by transport, leisure physical activity and sitting time by socioeconomic level from ELANS study

Country	Low		Medium		High		*P value*
	Mean (95% CI)	Median (Q1-Q3)	Mean (95% CI)	Median (Q1-Q3)	Mean (95% CI)	Median (Q1-Q3)	
Transport physical activity (min/week)							
Argentina	255.3 (224.9–287.6)	140.0 (60.0–300.0)	240.1 (210.5–268.5)	140.0 (60.0–280.0)	324.8 (214.7–442.3)	155.0 (70.0–420.0)	0.779
Brazil	221.4 (199.7–244.2)	120.0 (60.0–240.0)	193.6 (174.1–215.1)	105.0 (60.0–210.0)	180.3 (130.2–238.0)	100.0 (45.0–210.0)	0.149
Chile	206.4 (177.6–235.7)	105.0 (60.0–228.0)	221.7 (187.3–259.2)	120.0 (60.0–210.0)	246.6 (167.2–337.5)	105.0 (60.0–210.0)	0.763
Colombia	219.7 (197.8–242.0)	111.5 (60.0–210.0)	250.2 (213.2–290.6)	105.0 (60.0–280.0)	258.1 (181.7–355.6)	140.0 (87.0–329.0)	0.356
Costa Rica	269.2 (227.2–317.2)	140.0 (60.0–300.0)	284.7 (249.3–319.6)	168.5 (70.0–360.0)	253.1 (187.8–326.0)	140.0 (70.0–300.0)	0.338
Ecuador	227.5 (200.8–255.5)	140.0 (75.0–243.7)	216.6 (185.6–249.0)	120.0 (75.0–210.0)	210.3 (155.6–267.3)	140.0 (75.0–243.7)	0.300
Peru	201.5 (180.9–225.9)	120.0 (60.0–210.0)	169.7 (149.7–193.5)	105.0 (60.0–210.0)	213.3 (182.5–247.5)	140.0 (70.0–240.0)	0.169
Venezuela	174.2 (155.2–195.4)	100.0 (50.0–180.0)	195.7 (147.8–251.4)	105.0 (60.0–190.0)	170.7 (114.2–240.8)	95.0 (60.0–210.0)	0.618
Overall	218.1 (208.7–227.4)	120.0 (60.0–240.0)	222.2 (211.2–233.2)	120.0 (60.0–240.0)	225.2 (203.5–246.9)	120.0 (60.0–238.0)	0.471
Leisure physical activity (min/week)							
Argentina	312.9 (270.2–358.9)	200.0 (90.0–390.0)	281.8 (249.1–317.3)	180.0 (80.0–365.0)	316.5 (217.4–438.8)	225.0 (112.5–444.0)	0.608
Brazil	293.0 (252.4–336.3)	140.0 (60.0–360.0)	332.8 (295.4–371.9)	230.0 (87.5–477.5)	307.6 (239.1–381.9)	180.0 (90.0–442.5)	0.003
Chile	323.0 (277.4–371.5)	190.0 (90.0–420.0)	340.9 (291.7–393.3)	210.0 (76.2–420.0)	411.3 (304.7–512.4)	300.0 (120.0–438.7)	0.346
Colombia	286.2 (252.9–325.5)	143.5 (60.0–360.0)	310.9 (264.5–358.3)	180.0 (80.0–406.0)	235.8 (178.3–303.1)	195.0 (40.0–303.7)	0.252
Costa Rica	302.7 (243.4–366.3)	180.0 (62.5–360.0)	294.0 (248.2–342.3)	180.0 (80.0–360.0)	270.6 (202.2–346.7)	138.0 (60.0–375.0)	0.360
Ecuador	418.9 (371.8–468.6)	270.0 (140.0–540.0)	375.7 (327.5–430.0)	240.0 (120.0–440.0)	408.9 (320.0–499.3)	240.0 (150.0–507.5)	0.314
Peru	270.2 (235.0–312.2)	139.0 (60.0–330.0)	268.7 (226.6–312.3)	155.0 (60.0–360.0)	281.6 (234.5–333.1)	200.0 (75.0–420.0)	0.035
Venezuela	344.7 (299.5–391.2)	240.0 (120.0–420.0)	313.9 (220.2–405.6)	180.0 (60.0–472.5)	246.2 (142.8–363.9)	120.0 (56.0–480.0)	0.466
Overall	316.8 (300.7–316.9)	180.0 (80.0–405.0)	315.1 (298.6–331.7)	180.0 (90.0–416.0)	317.9 (288.4–347.4)	210.0 (90.0–420.0)	0.081
Sitting time (min/day)							
Argentina	539.5 (515.6–563.4)	480.0 (330.0–720.0)	544.0 (519.3–568.4)	510.0 (352.5–720.0)	522.6 (452.6–589.8)	495.0 (240.0–750)	0.920
Brazil	401.5 (382.6–420.8)	360.0 (180.0–558.0)	453.9 (433.9–474.5)	390.0 (240.0–600.0)	518.9 (467.3–572.5)	360.0 (180.0–558.0)	<0.001
Chile	457.7 (430.3–484.2)	420.0 (240.0–600.0)	506.5 (477.6–534.3)	480.0 (300.0–660.0)	532.2 (474.5–592.9)	480.0 (360.0–660.0)	0.026
Colombia	474.1 (451.2–498.9)	420.0 (240.0–660.0)	497.8 (466.4–528.4)	440.0 (290.0–660.0)	479.5 (417.9–549.1)	480.0 (300.0–600.0)	0.710
Costa Rica	405.1 (371.6–438.8)	360.0 (180.0–540.0)	480.1 (451.1–512.0)	420.0 (240.0–660.0)	457.5 (394.1–523.3)	360.0 (180.0–660.0)	0.140
Ecuador	331.1 (302.6–358.3)	265.0 (150.0–480.0)	355.8 (329.2–381.4)	300.0 (180.0–480.0)	362.6 (316.7–414.1)	360.0 (180.0–480.0)	0.185
Peru	515.9 (488.6–540.2)	480.0 (300.0–690.0)	515.6 (483.9–546.6)	480.0 (330.0–660.0)	565.0 (529.8–599.2)	525.0 (360.0–712.5)	0.139
Venezuela	377.9 (360.4–394.9)	360.0 (180.0–540.0)	412.8 (371.4–451.2)	390.0 (195.0–600.0)	367.3 (295.5–444.5)	315.0 (180.0–480.0)	0.293
Overall	428.1 (415.9–440.3)	370.0 (210.0–600.0)	471.5 (457.7–485.2)	420.0 (240.0–660.0)	490.9 (466.0–515.7)	477.5 (300.0–660.0)	<0.001

95% CI: 95% confidence interval

**Table 5:** Characteristics of participants by transport, leisure physical activity and sitting time by education level from ELANS study

Country	Low		Medium		High		*P value*
	Mean (95% CI)	Median (Q1-Q3)	Mean (95% CI)	Median (Q1-Q3)	Mean (95% CI)	Median (Q1-Q3)	
Transport physical activity (min/week)							
Argentina	265.5 (238.6–292.4)	140.0 (60.0–300.0)	221.9 (185.4–258.5)	120.0 (60.0–280.0)	175.1 (120.8–229.4)	140.0 (75.0–210.0)	0.595
Brazil	218.2 (195.3–241.1)	107.5 (60.0–213.7)	190.2 (171.8–208.6)	105.0 (60.0–210.0)	216.8 (158.9–274.7)	135.0 (50.0–217.5)	0.644
Chile	205.5 (179.9–231.0)	105.0 (60.0–212.5)	218.1 (171.6–264.7)	120.0 (60.0–213.7)	281.2 (186.3–376.1)	140.0 (60.0–315.0)	0.267
Colombia	242.8 (217.9–267.7)	120.0 (60.0–276.0)	199.4 (163.9–234.8)	105.0 (49.7–210.0)	228.0 (173.9–282.1)	120.0 (60.0–240.0)	0.058
Costa Rica	276.5 (248.5–304.5)	157.5 (60.0–346.2)	260.7 (189.4–331.9)	120.0 (70.0–290.0)	289.6 (184.2–394.9)	140.0 (75.0–390.0)	0.451
Ecuador	226.7 (204.9–248.5)	140.0 (75.0–240.0)	174.2 (113.3–235.2)	100.0 (60.0–150.0)	231.6 (148.0–315.1)	120.0 (70.0–210.0)	0.009
Peru	193.3 (163.1–223.6)	107.5 (60.0–210.0)	195.9 (177.3–214.6)	120.0 (60.0–210.0)	186.9 (150.7–223.3)	120.0 (73.7–210.0)	0.590
Venezuela	175.9 (154.3–197.6)	90.0 (50.0–180.0)	196.0 (144.9–247.2)	105.0 (60.0–210.0)	170.3 (126.4–214.3)	100.0 (60.0–168.7)	0.523
Overall	230.5 (221.4–239.6)	120.0. (60.0–250.0)	200.7 (189.8–211.6)	110.0 (60.0–210.0)	215.9 (193.8–238.1)	120.0 (60.0–210.0)	0.103
Leisure physical activity (min/week)							
Argentina	297.0 (263.3–330.7)	180.0 (90.0–360.0)	309.4 (264.9–353.9)	240.0 (120.0–405.0)	265.9 (147.7–384.3)	180.0 (90.0–360.0)	0.019
Brazil	321.6 (281.3–361.9)	170.0 (72.5–420.0)	302.8 (262.3–343.3)	180.0 (60.0–427.5)	311.5 (247.2–375.8)	240.0 (90.0–455.0)	0.391
Chile	332.1 (289.4–374.8)	205.0 (90.0–420.0)	326.9 (254.8–399.0)	210.0 (60.0–420.0)	413.0 (307.2–518.8)	300.0 (120.0–420.0)	0.159
Colombia	283.3 (247.5–319.1)	150.0 (60.0–300.0)	305.8 (257.5–354.2)	180.0 (60.0–420.0)	298.3 (219.9–376.6)	150.0 (60.0–335.0)	0.273
Costa Rica	288.8 (250.8–326.7)	170.0 (60.0–360.0)	309.5 (212.7–406.2)	180.0 (90.0–360.0)	311.5 (201.1–421.9)	207.5 (108.7–363.7)	0.147
Ecuador	406.2 (371.4–441.1)	255.0 (127.5–527.5)	358.6 (263.9–453.1)	217.5 (120.0–425.0)	410.6 (244.8–576.4)	255.0 (145.0–447.5)	0.473
Peru	289.1 (229.7–348.5)	150.0 (60.0–340.0)	274.6 (245.3–303.9)	160.0 (61.0–360.0)	218.5 (150.8–286.2)	140.0 (60.0–240.0)	0.643
Venezuela	348.6 (294.2–403.0)	210.0 (105.0–435.0)	242.2 (185.4–298.9)	240.0 (67.5–345.0)	350.1 (268.7–431.5)	240.0 (67.5–345.0)	0.427
Overall	325.2 (310.8–339.5)	180.0 (90.0–420.0)	297.7 (279.8–315.5)	180.0 (75.0–391.2)	320.8 (287.9–353.6)	202.5 (90.0–403.7)	0.353
Sitting time (min/day)							
Argentina	532.6 (513.9–551.2)	480.0 (300.0–720.0)	563.9 (529.8–597.9)	540.0 (360.0–720.0)	574.8 (485.6–663.9)	540.0 (300.0–735.0)	0.243
Brazil	398.1 (379.2–416.9)	360.0 (180.0–540.0)	458.2 (436.6–479-8)	420.0 (240.0–640.0)	528.1 (480.8–575.4)	480.0 (360.0–720.0)	<0.001
Chile	466.4 (443.2–489.6)	420.0 (270.0–600.0)	532.6 (491.573.6)	480.0 (340.0–720.0)	499.8 (449.5–550.0)	420.0 (360.0–600.0)	0.082
Colombia	455.4 (433.3–477.6)	420.0 (220.0–655.0)	530.6 (494.7–566.5)	480.0 (300.0–720.0.)	529.7 (477.7–581.8)	510.0 (300.0–720.0)	<0.001
Costa Rica	432.8 (409.9–455.7)	360.0 (240.0–600.0)	518.6 (450.2–586.9)	450.0 (260.0–720.0)	579.5 (479.2–679.8)	540.0 (360.0–780.0)	0.005
Ecuador	333.1 (314.2–352.0)	270.0 (160.0–480.0)	416.0 (356.9–475.1)	360.0. (240.0–510.0)	370.6 (310.2–430.9)	360.0 (213.7–540.0)	0.005
Peru	529.6 (491.5–567.7)	480.0 (300.0–710.0)	519.5 (498.7–540.4)	480.0 (315.0–660.0)	560.6 (514.6–606.7)	540.0 (382.5–712.5)	0.080
Venezuela	376.1 (356.9–395.3)	360.0 (180.0–500.0)	355.1 (312.5–397.8)	315.0 (180.0–485.0)	429.9 (394.0–465.8)	420.0 (240.0–600.0)	0.001
Overall	436.5 (428.7–444.3)	370.0 (225.0–600.0)	495.7 (484.2–507.3)	456.0 (270.0–660.0)	503.7 (484.5–522.8)	480.0 (300.0–660.0)	<0.001

95% CI: 95% confidence interval

Overall, the prevalence of insufficient physical activity in the transport and leisure domains were 69.9% (95% CI: 68.9–70.8) and 72.8 (95% CI: 72.0–73.7); ranging from 59.8% (Chile) to 81.0% (Venezuela) for transport and 46.1% (Ecuador) to 83.8% (Venezuela) for leisure (Additional file 1: Figure S1). In each country and overall, women (76.9; 95% CI: 75.8–78.1) were more likely to be insufficient physical activity than men (68.4%; 95% CI: 67.0–69.8) for leisure (Additional file 2: Figure S2).

Overall, insufficient transport physical activity prevalence was lower among those aged 30–59 years (68.7%; 95% CI: 67.4–73.0) compared to those aged <30 years (71.5%; 95% CI: 69.9–73.0) and those aged ≥60 years (69.1%; 95% CI: 65.5–72.5). Insufficient leisure physical activity prevalence was lower among those aged < 30 years compared to those aged 30–59 and ≥ 60 years (Additional file 3: Figure S3).

Participants with low socioeconomic level and low education level had a slightly higher percentage of insufficient physical activity. However, these trends were not observed in all countries for transport physical activity. Persons of high socioeconomic level (65.8%; 95% CI: 62.6–68.6) had a lower prevalence of insufficient leisure physical activity overall compared with those of middle socioeconomic level (75.2%; 95% CI: 73.9–76.4) or low SES (71.4%; 95% CI: 69.9–73.0) (Additional file 4: Figure S4). Individuals with low (74.1%; 95% CI: 73.0–75.3) or middle (72.0%; 95% CI: 70.2–73.6) education levels had a higher prevalence of insufficient leisure physical activity compared with those with a high education level (67.4%; 95% CI: 64.2–70.6) (Additional file 5: Figure S5).

## Discussion

The aim of this study was to quantify and characterise socio-demographic patterns of physical activity and sitting time in eight Latin American countries. On average, participants spent 220.3 min/week (median: 120.0 min/week) in transport physical activity, 316.4 min/week (median: 180.0 min/week) in leisure physical activity and 460.2 min/day (median: 420.0 min/day) in sitting time. When all countries were analyzed together, transport and leisure physical activity and sitting time were higher in men than women. The mean and median of transport physical activity were similar across age groups, but leisure physical activity was higher in the 15–29 group than for those 30–59 and ≥ 60 years old in Brazil (*p* = 0.011), Venezuela (*p* = 0.001) and overall (*p* < 0.001). Sitting time was highest among those with higher socioeconomic and education levels. In contrast, the relationships between physical activity and socioeconomic and education levels were more variable across countries.

The present study reports population-level prevalence estimates and patterns of physical activity and sitting time in urban samples from eight countries using a comparable, reliable, and validated survey instrument [22]. Previous similar studies in Latin America have generally assessed physical activity at the sub-national level [33–37] and have not used standard surveys, timelines, and methods in representative national samples [37]. In contrast, ELANS was conducted simultaneously in the urban populations of the most populous cities of eight countries in Latin America. Despite the many manuscripts describing the global impacts of physical inactivity [4, 5, 10, 38] and global calls for action to reverse the physical inactivity pandemic, few physical activity interventions have occurred in Latin America. While cross-country interventions may be challenging given varying cultural, geographical, social, and economic milieus in different countries, the current results suggest some similarities that may set the stage for further exploration and intervention development [39, 40].

Compared with the rest of the world, Latin American countries had high prevalences of insufficient physical activity (i.e. not meeting WHO guidelines) [41]. Our analyses show that the prevalence of insufficient transport and leisure physical activity varies greatly across the eight Latin American countries (Fig. S1-S5); insufficient physical activity was lower in Costa Rica (59.8%) and higher in Venezuela (81.0%) for transport and Ecuador (46.1%) and Venezuela (83.8%) for leisure. Werneck et al. [34] compiled self-reported data from six surveys across South American countries (116.982 participants) showed that the highest levels of leisure physical inactivity (< 150 min/week) were in Peru (91.4%), Ecuador (84.7%), Brazil (79.7%), Chile (79.2%) and Argentina (70.8%). Besides the leisure physical activity, active transportation has beneficial effects on all-cause mortality, cardiovascular disease and cancer and can increase the physical activity levels of entire populations [12, 42, 43]. Weekly transport physical activity time was highest in Costa Rica (mean: 275.3 min/week; median: 147.0 min/week) and lowest in Venezuela (mean: 177.6 min/week; median: 100.0 min/week). Active transportation (walking and cycling) is potentially an important contributor to health, particularly in highly urbanized regions like Latin America [39, 44], in which it may improve people’s mental and physical health, prevent road traffic-related injury, and decrease environmental pollution [43].

The presence of “ciclovías” improved the participation of adults in active transportation by walking [45]. Results from such programs are important because they can support the actions described in the new urban plans of several countries from Latin America (i.e., Peru, Santiago, Colombia, and Brazil). These plans include efforts aimed at increasing accessibility to public parks [46]. For example, The “Ciclovía-Recreativa” programs from Bogotá have shown that users of “ciclovía programmes” contribute substantially to meeting physical activity guidelines and have better quality of life [47]. Such public health campaigns can inspire populations to travel by walk and cycle more [48]. “Ciclovías”, which temporarily close streets to private transport to create a safe place for people to cycle, walk, run, and participate in social health promotion and cultural events, have been shown to be very effective “programmes” in the Latin American region [39].

Sex differences in transport and leisure physical activity have been reported in studies from countries with different income levels [41]. Overall, our investigation found, similarly to other regions (Mexico, Europe, and the United States) [49–51], significantly lower physical activity in women than men. Such results argue for interventions targeting women to help close the sex difference and reach the global physical activity goals [41, 52]. Of note, how women, vs. men respond to their local built environments, including the walkability of their environment, has been identified as a major correlate of physical activity levels worldwide [53]. A better understanding of sex differences can also occur through measuring their participation in diverse domains of activity (i.e., transport and leisure time activities). More opportunities for safe and available leisure activities for women, as well as the impacts of cultural norms, traditional roles, and lack of social and community support all can lead to reduced participation in physical activities among women [52]. Understanding and addressing these barriers are necessary to plan and deliver socially sensitive programs to support behavior change. Another way to improve leisure-time physical activity may be to promote women’s involvement in sport, as women do not have the same opportunity to engage in sport. Collaboration from the government, sports institutions and health professionals can help to increase women’s participation in physical activity.

Overall, physical activity (transport and leisure) and sitting time showed high variability across countries. There was, however, no clear pattern in the time spent in physical activity and sitting in relation to differences at the socioeconomic and education levels. The current patterns of physical activity and sitting time by socioeconomic strata are closely related to urban development in Latin America characterized by social and environmental inequalities, unplanned and disorganized growth, and underlying political and socioeconomic factors [54, 55]. Factors such as globalization and industrialization influenced the migration from rural to urban areas. The fact that physical activity and sitting time vary greatly across countries and cities suggests that the factors that influence inactivity lie at national, sub-national, and community levels, and policies specific to these levels may be needed to increase physical activity [4].

The differences in physical activity with socioeconomic level are clearer when evaluated at the level of the transport and leisure domains [56]. We found differences between socioeconomic level strata and leisure physical activity in Brazil and Peru. There have been reports of stronger relationship between socioeconomic strata of leisure physical activity in European countries [56]. A higher socioeconomic position is associated with better facilities and environments and more opportunities for leisure time physical activity [35]. Building more places appropriate for leisure time physical activity such as parks outdoor courts and bicycle paths [35] and improving walkability of streets [33] may be important strategies for increasing opportunities for leisure physical activity among lower SES groups in Latin America. These actions are included in national physical activity policies in Latin America countries [57, 58].

Each country in our study had a mean level of sitting time higher than 7 hours/day. Van Dyck et al. [59] reported means of 7.9 and 7.8 h/day of sedentary behaviour in Brazil and Colombia. Most countries in the current study showed socioeconomic (Brazil, Chile, Ecuador, Peru, overall) and educational (Argentina, Brazil, Colombia, Costa Rica, Peru, Venezuela and overall) gradients in sitting time, with higher levels with higher socioeconomic and education level. Presumably adults with higher education and from higher income groups have more sedentary jobs, are more likely to use cars than active travel as a means of transport, and have more electronic entertainment and labor-saving devices at home. Cultural factors may also explain some patterns, through behavioral preferences [60].

Our study showed that in most countries, younger participants (<30 years) spent more time sitting time than older participants (≥30 years). This behaviour indicates more frequent sedentary occupations in the use of passive transport among younger adults. It is possible that this pattern could signal a future increase in for poor health outcomes [61]. While the need to monitor sedentary behaviours in national health surveys seems clear, few efforts exist to study sedentary behaviour worldwide [34], and no policies to decrease sedentary behaviours exist in Latin America.

ELANS provides data allowing comparisons across eight countries from Latin American for the first time. Major inputs included the production of comparable physical activity values in eight countries using a common protocol. Many manuscripts showed moderate correlations between IPAQ and accelerometers [62, 63]. Questionnaires remain the most practical method for measuring physical activity in populations due to the low price and high burden of respondents [64]. Compared with many current physical activity questionnaires, a strength of IPAQ is the ability to quantify both leisure and transport physical activity. IPAQ is widely used for measuring and tracking physical activity levels in Latin American populations [23, 36, 65]. Its use in Latin America has not been without challenges, and has required several cultural and structural adaptations. IPAQ measurement results can be overestimated [66–68]. Total physical activity may have higher values than only leisure activities [69]. This between-country difference appeared even greater in the low- and middle- income countries [70]. Another limitation in this study was the complexity of socioeconomic strata classification that may have led to misclassification within the three socioeconomic levels. Developing a feasible, realistic, standardized socioeconomic strata categorization was more difficult than expected, requiring extensive and innovative work. Measurement and definitions of socioeconomic status and educational level across countries requires close attention to ensure comparability [33, 56].

## Conclusions

The study findings show wide variation in transport and leisure physical activity and sitting time by sex and age group in eight Latin America countries. The results do not show significant difference in transport and leisure physical activity by socioeconomic and education levels. The observed variability across countries sets the stage for future investigations to inform interventions at the national and regional levels. Future studies should seek to better understand the challenges of promoting transport and leisure physical activity and reducing sitting time in urban regions. Such studies may help in gaining a deeper understanding of the factors that can be targeted to increase physical activity in Latin America.

## Supplementary information

**Additional file 1: Figure S1.** Prevalence (% and 95 confidence interval) of insufficient physical activity from eight Latin America countries.

**Additional file 2: Figure S2.** Prevalence (% and 95 confidence interval) of insufficient physical activity by sex from eight Latin America countries.

**Additional file 3: Figure S3.** Prevalence (% and 95 confidence interval) of insufficient physical activity by age group from eight Latin America countries.

**Additional file 4: Figure S4.** Prevalence (% and 95 confidence interval) of insufficient physical activity by socioeconomic level from eight Latin America countries.

**Additional file 5: Figure S5.** Prevalence (% and 95 confidence interval) of insufficient physical activity by education level from eight Latin America countries.

## Data Availability

The dataset used and analysed during the current study are available from the corresponding author on reasonable request.

## Abbreviations

CI95%: 95% confidence interval
CVD: Cardiovascular diseases
ELANS: Latin American Study of Nutrition and Health / Estudio Latinoamericano de Nutrición y Salud
IPAQ: International Physical Activity Questionnaire
min: minutes
PA: Physical activity
ST: Sitting time
WHO: World Health Organization
