# Supplementary figures and images for: Original research Socio-demographic patterning of self-reported physical activity and sitting time in Latin American countries: findings from ELANS
Source: BMC Public Health. 2019 Dec 23;19:1723. doi: 10.1186/s12889-019-8048-7 (PMC6929436; doi:10.1186/s12889-019-8048-7)

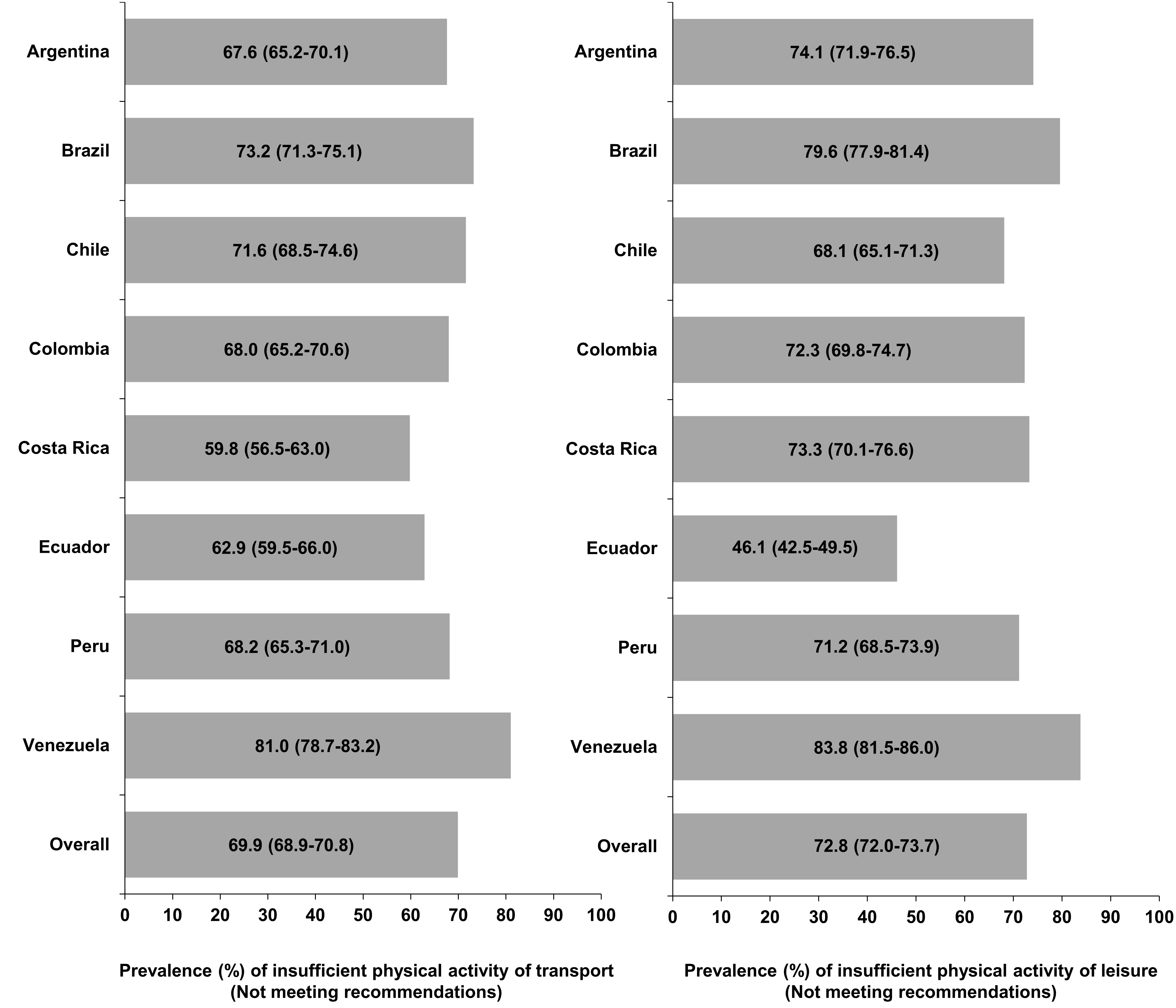

Supplement: Supplementary file 1 — Additional file 1: Figure S1. Prevalence (% and 95 confidence interval) of insufficient physical activity from eight Latin America countries. [file 12889_2019_8048_MOESM1_ESM.jpg]

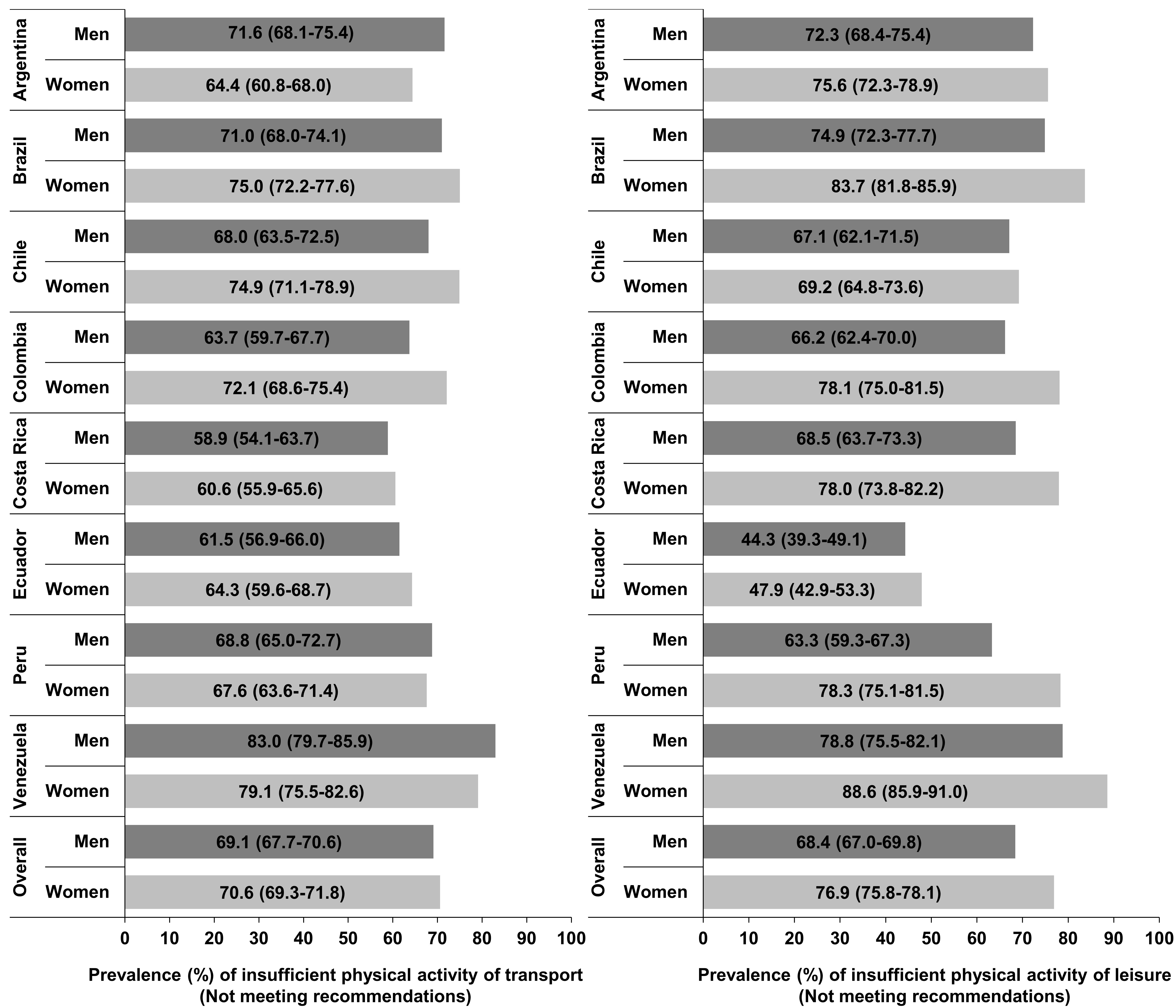

Supplement: Supplementary file 2 — Additional file 2: Figure S2. Prevalence (% and 95 confidence interval) of insufficient physical activity by sex from eight Latin America countries. [file 12889_2019_8048_MOESM2_ESM.jpg]

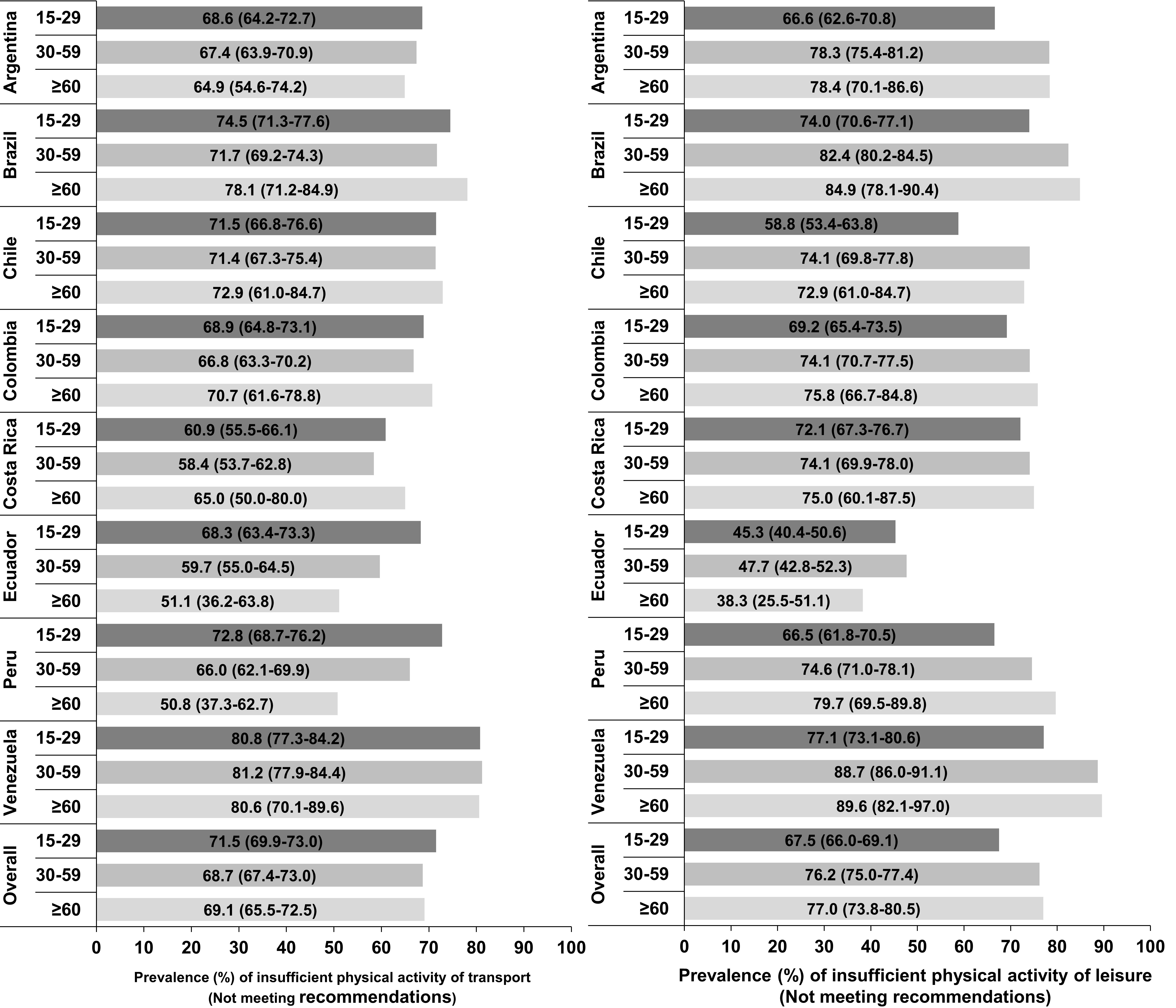

Supplement: Supplementary file 3 — Additional file 3: Figure S3. Prevalence (% and 95 confidence interval) of insufficient physical activity by age group from eight Latin America countries. [file 12889_2019_8048_MOESM3_ESM.jpg]

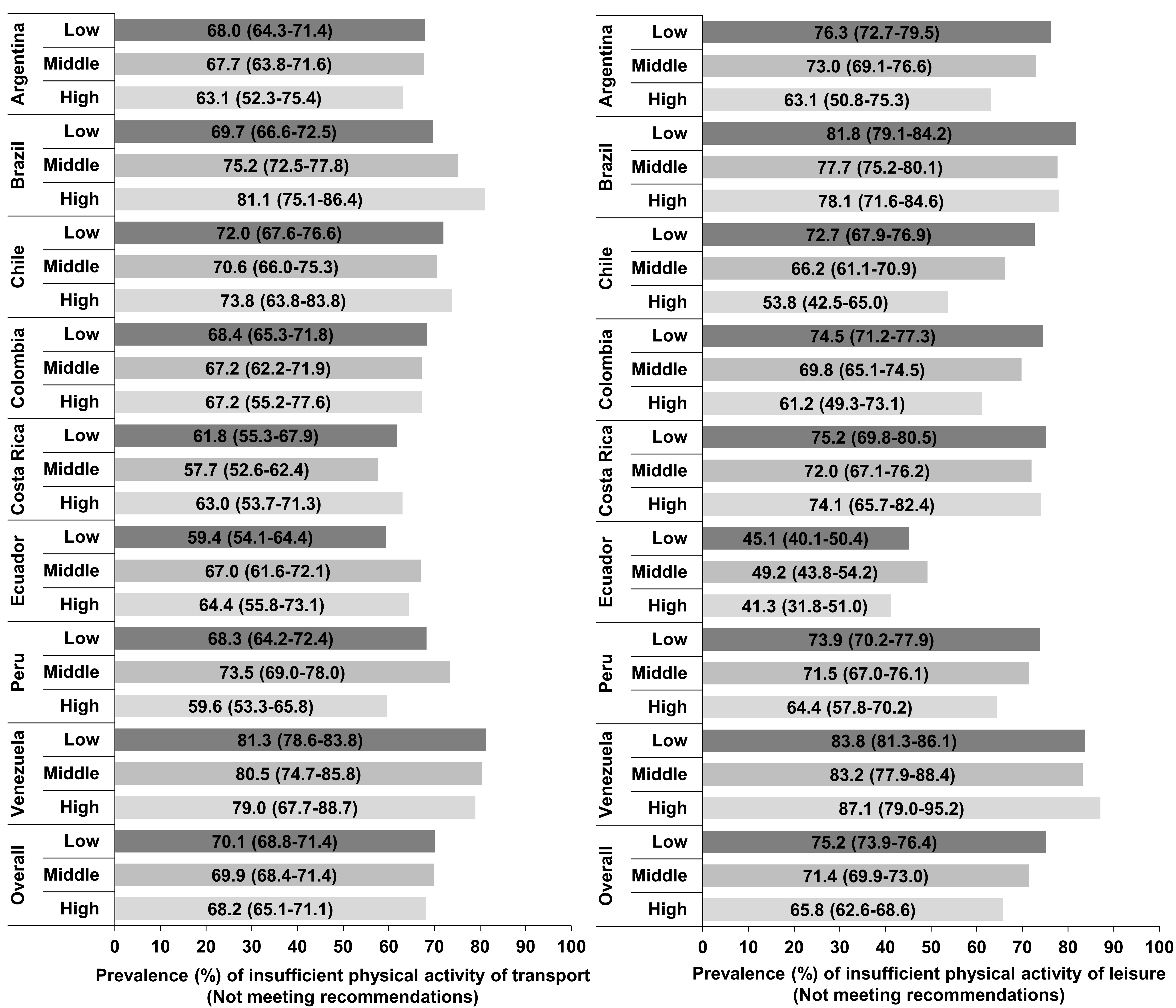

Supplement: Supplementary file 4 — Additional file 4: Figure S4. Prevalence (% and 95 confidence interval) of insufficient physical activity by socioeconomic level from eight Latin America countries. [file 12889_2019_8048_MOESM4_ESM.jpg]

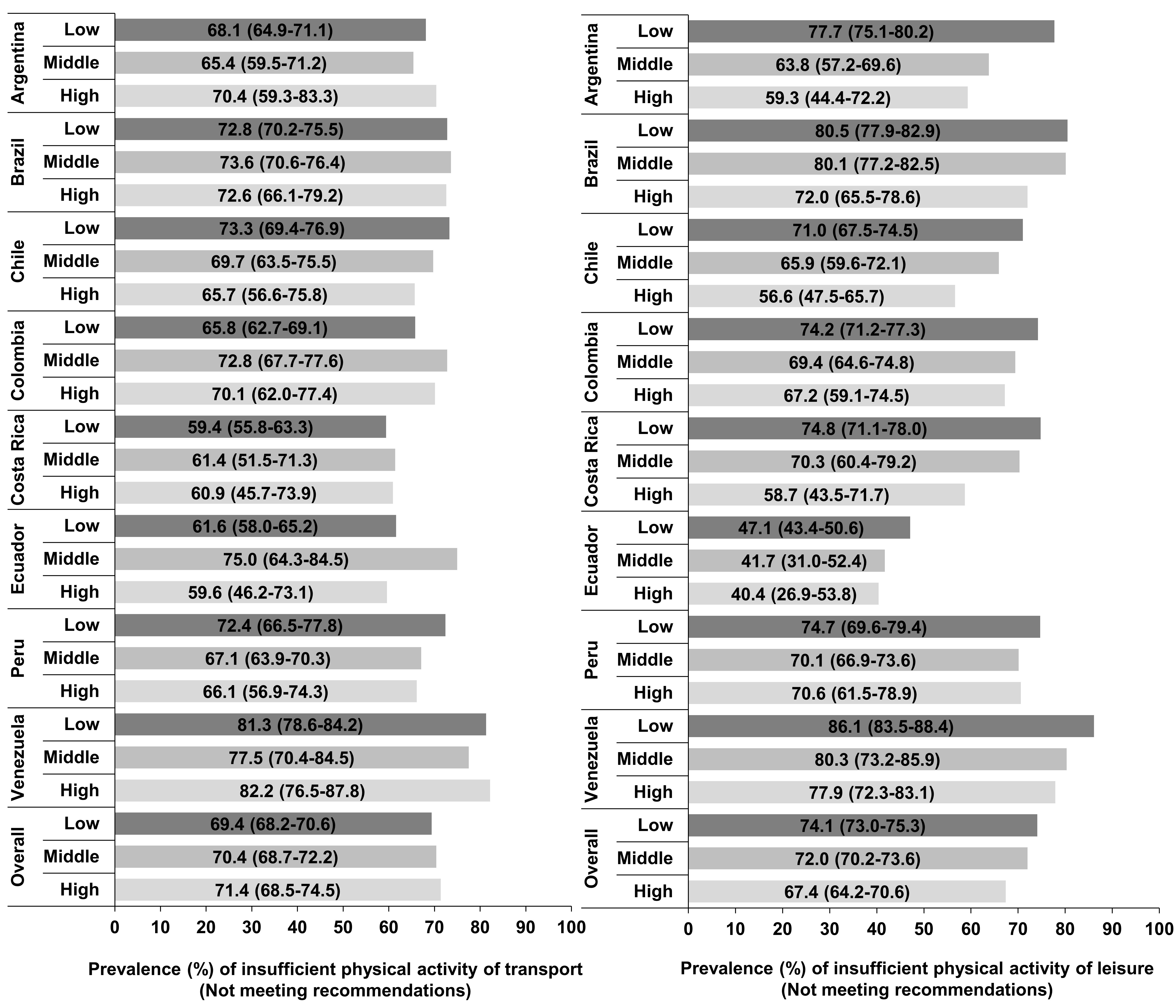

Supplement: Supplementary file 5 — Additional file 5: Figure S5. Prevalence (% and 95 confidence interval) of insufficient physical activity by education level from eight Latin America countries. [file 12889_2019_8048_MOESM5_ESM.jpg]
